# A new quality assurance package for hospital palliative care teams: the Trent Hospice Audit Group model

**DOI:** 10.1038/sj.bjc.6601945

**Published:** 2004-06-22

**Authors:** J Hunt, V L Keeley, M Cobb, S H Ahmedzai

**Affiliations:** 1 Academic Palliative Medicine Unit, Clinical Sciences Division (South), The University of Sheffield, Royal Hallamshire Hospital, Sheffield S10 2JF, UK; 2 Nightingale Macmillan Unit, Derbyshire Royal Infirmary, Derby DE1 2QS, UK; 3 Sheffield Teaching Hospitals NHS Trust, Royal Hallamshire Hospital, Sheffield S10 2JF, UK

**Keywords:** palliative care, quality assurance, audit, standards, hospital teams

## Abstract

Cancer patients in hospitals are increasingly cared for jointly by palliative care teams, as well as oncologists and surgeons. There has been a considerable growth in the number and range of hospital palliative care teams (HPCTs) in the United Kingdom. HPCTs can include specialist doctors and nurses, social workers, chaplains, allied health professionals and pharmacists. Some teams work closely with existing cancer multidisciplinary teams (MDTs) while others are less well integrated. Quality assurance and clinical governance requirements have an impact on the monitoring of such teams, but so far there is no standardised way of measuring the amount and quality of HPCTs' workload. Trent Hospice Audit Group (THAG) is a multiprofessional research group, which has been developing standards and audit tools for palliative care since the 1990s. These follow a format of structure–process–outcome for standards and measures. We describe a collaborative programme of work with HPCTs that has led to a new set of standards and audit tools. Nine HPCTs participated in three rounds of consultation, piloting and modification of standard statements and tools. The final pack of HPCT quality assurance tools covers: policies and documentation; medical notes review; questionnaires for ward-based staff. The tools measure the HPCT workload and casemix; the views of ward-based staff on the supportive role of the HPCT and the effectiveness of HPCT education programmes, particularly in changing practice. The THAG HPCT quality assurance pack is now available for use in cancer peer review.

Specialist palliative care has, since the 1980s, expanded its services in order to offer direct care and support to people living at home and to those in acute hospital beds and outpatient clinics.

The Calman-Hine report (1995) emphasised the requirement for the provision of palliative care throughout cancer care: ‘there should be a smooth progression of [palliative] care between home, hospital and hospice’ (p 16). The National Council for Hospice and Specialist Palliative Care Services (NCHSPCS) Occasional Paper 10 ‘Palliative Care in the Hospital Setting’ (1996) stated that

A hospital palliative care team provides specialist palliative care within the acute hospital setting. The team has an advisory and educational role and may also provide direct care to patients and their families requiring a high level of palliative care skills (p 7).

A later NCHSPCS document ‘Palliative Care 2000’, (1999) identified the core and extended members of a hospital palliative care team as

One or more nurses who hold or who are working towards a specialist practitioner recordable qualification in palliative care; a consultant in palliative medicine supported by other medical staff including junior staff who may be on rotations.

Secretarial/administrative support. The extended team should include chaplaincy, social work, psychology and pharmacy expertise and access to specialist pain management. In addition, there should be access to physiotherapy, occupational therapy and dietetics.' (p 41)

HPCTs have since grown to include dedicated social workers, chaplains, allied health professionals and pharmacists. The emergence of cancer site-specific multidisciplinary core and extended teams has seen a greater specialisation for many palliative care clinical nurse specialists (CNSs) and a further involvement in joint clinic activity for medical members of the team.

The Clinical Governance agenda for the NHS (adapted, but with the same component requirements, for independent hospice services) has impacted upon HPCTs, like all other palliative care service providers. This has included a rigorous review of systems, policies and procedures, documentation and the adoption of appropriate quality measures to assist the evidence base for clinical effectiveness (NCHSPCS, 2000).

Measures used to date have focussed on workload/casemix and the effectiveness of interventions.

## 

### Workload

The ‘levels of intervention’ first described by Webber (1994) range from telephone advice to regular visiting of a patient to manage, monitor and reassess. This has become the standard method of allocating and recording workload among teams.

### Effectiveness of interventions

Quality measures have been used by HPCTs to monitor the effectiveness of interventions. The Support Team Assessment – STAS (Higginson, 1993) (modified for hospital use from a tool developed for use in the community) and its ‘daughter’ measure, the Palliative care Outcome Scale – POS (Hearn and Higginson, 1999) have both figured as tools. A modification of STAS, E-STAS has also been established ‘as a useful tool to evaluate interventions by a hospital palliative care team’ (Edmonds *et al*., 1998).

Ellershaw *et al*. (1995) developed the patient care assessment form (PACA) specifically to measure the effectiveness of a hospital palliative care team ‘in the provision of symptom control, patients' and relatives' awareness of the diagnosis, and outcome regarding the patients' placements’.

To date the only standards developed for HPCTs are in the NHS Manual of Cancer Standards (2000). These are limited to a defined membership of the core and extended team (with qualifications for CNS), requirements for regular clinical meetings to discuss patients, regular meetings to discuss operational issues, the availability of the team ‘out of hours’, the production of a service directory, the existence of a palliative care strategy group and for clinical audits to be carried out by the team.

The aim of the work described in this paper was to develop (and validate) a comprehensive set of standards for HPCTs together with audit tools to monitor compliance.

## TRENT HOSPICE AUDIT GROUP MODEL

The Trent Hospice Audit Group (THAG) was formed in 1990 as a working group of senior physicians and nurses from specialist palliative care in the former Trent Region of the UK. It has produced ‘Palliative Care Core Standards’ for inpatient palliative care services (Ahmedzai *et al*, 1998) based on the Donabedian framework of Structure–Process–Outcome (S–P–O) criteria, with audit tools (Box 1

**Box 1:** Donabedian's structure–process–outcome criteria

**Structure, process and outcome**
***Definitions***
*Structure*
The *inputs* to care: staff and resources, including the physical and organisational settings in which the service is provided. Structure is relatively easy to measure, for example, staff numbers, beds, size of budget, but its relationship to quality of outcome (see below) is uncertain
*Process*
The *activities* of providing care, which uses resources and produces outcomes. Measuring process can include both *what* is done (e.g. counting staff–patient contacts) and *how* it is done (e.g. what procedures are followed)
*Outcome*
The result of the clinical intervention, as reflected in the patient's health status and quality of life

^*^Adapted from, National Council for Hospice and Specialist Palliative Care Services (1997) Making Palliative Care Better, p 1.0.

).

Since 2001, THAG has concentrated on broadening its quality measures to incorporate palliative care services in the community and acute hospitals. These developments have incorporated the views of a wide range of palliative care service providers, the quality measure ‘users’.

This paper describes the development of a Hospital Palliative Care Team standard and audit package using the THAG approach.

## METHODS

The THAG Hospital Palliative Care Team Standard was initially developed in 2001 by an *ad hoc* panel of consultants in palliative medicine and clinical nurse specialists, responding to a call for those interested in producing a quality measure. The panel used policy and planning documents produced by the NCHSPCS as reference.

The main aims of the standard development group were:

- To produce a quality measure for specialist hospital teams.
- To audit not only the effectiveness of the HPCTs' systems, but also its impact, on patient needs, on ward team staff and on the development of knowledge, skills and practice within an acute hospital setting.
- To enable palliative care teams and services to develop an acute hospital service using the standard as guidance.

As described earlier, criteria were developed using the S–P–O format. Box 2

**Box 2:** THAG hospital palliative care team – standard statement and examples of structure, process and outcome criteria

**Standard statement***:*
The Hospital Palliative Care Team is an effective resource to referring hospital specialties, patients, their carers/families and staff

*Structural criteria: (examples only)*
S2: The team has an operational policy relating to its clinical role and function
S9: The team has written guidelines for the control of pain and other distressing symptoms, for ward-based teams
S11: The HPCT has a system for maintaining records of referrals, discharges/deaths, details of counselling and bereavement support to families/carers and quick reference on medication, pain control and other key clinical information

*Process criteria: (examples only)*
P1: HPCT members undertake initial and ongoing, holistic assessment of the needs of the patient and their family carers
P3: All aspects of HPCT intervention are recorded in the patients' medical notes
P9: The HPCT provides informal and formal multidisciplinary education throughout the hospital

*Outcome criteria: (examples only)*
O1: Ward and other clinical teams state that the HPCT has assisted effectively with the management of patients with chronic progressive illnesses
O3: A validated evaluation package demonstrates the effectiveness of HPCT education in improving knowledge, skills and practice of general palliative care in the hospital

contains the standard statement and examples of the Structure, Process and Outcome criteria.

The first draft package included:

- A documentation audit – designed to ensure that the HPCT had dated, signed operational policies and procedures; that it performed its resource function, with documented guidelines on pain and symptom control measures and evidence of educational programmes and that it recorded, in its own documentation, key aspects of intervention activity. (The auditor noted each recorded visit, the discipline of the HPCT member who made the visit and the nature of the visit; assessment, monitoring, symptom control advice, psychological support to patient and/or informal carer, discharge planning discussions or liaison with other professionals.)
- A questionnaire for ward staff – designed to elicit staff's views on the availability and effectiveness of the HPCT in supporting patients and families, in advising on symptom management and in delivering staff support. It also sought to identify the level of attendance at HPCT educational events during the previous 2 years.
- An education evaluation questionnaire – designed to enable ward-based staff to indicate the value of HPCT teaching in terms of new knowledge, new skills and change in practice.

Circulation for consultation followed. Teams were targeted using two criteria:

- Hospital based teams in Trent – including the consultants in palliative medicine and clinical nurse specialists who had participated in the development.
- Teams who had registered with THAG as users of other existing standards or interested services.

Four acute hospital trust HPCTs – two in Trent, one each from the North West and the South East – were identified as pilot sites for the revised draft (Table 1

**Table 1:** HPCT standard pilot site trusts

**Round 1**	**Round 2**
Place: University Hospitals of Leicester NHS Trust	Place: King's College Hospital NHS Trust
Type: Cancer Centre	Type: Cancer Centre
Place: Blackburn Hyndburn & Ribble Valley NHS Trust	Place: Queen's Medical Centre Nottingham University Hospital NHS Trust
Type: Cancer Unit	Type: Cancer Centre
Place: Southern Derbyshire Acute Hospitals NHS Trust	Place: Guy's and St Thomas' Hospital NHS Trust (2 sites)
Type: Cancer Centre	Type: Cancer Centre
Place: Thames Gateway NHS Trust	Place: St Helens and Knowsley Hospitals NHS Trust
Type: Cancer Unit	Type: Cancer Unit
	
Total sites in this round=4	Total sites in this round=5

). First pilot audits took place between January and March 2002. The same auditor (JH) undertook all pilot site visits.

The main purpose of the pilot audits was to test the usability, relevance and coverage of the audit tools. The HPCTs received a written report within 2 weeks of the audit exercise. The reports were consistently divided into three sections:

- The response to the evidence provided relating to the audit questions.
- Comments or suggestions, which the team might find useful, relating to some of the evidence material.
- Recommendations for changes or additions to the standard criteria or audit tool questions.

Following a pilot audit, further revisions were made and a second round of auditing took place during July and August 2002 using version 6. Five Trust HPCTs were visited. None was the same as in the first phase of piloting. The sites were in London (× 3), the North West and Trent (Table 1).

Guidance was offered to participating teams to assist their preparation. Each team identified a coordinator. In most cases this was a CNS but a Specialist Registrar in Palliative Medicine and an Administrator also undertook that role. The documentary evidence was collected into a folders or folders, titled or divided into S–P–O sections. The coordinator facilitated visits to the wards (selected by the site's HPCT) and introduced the auditor. Recruitment of staff for interview was undertaken by the ward manager. Visit times were at the convenience of the ward. The coordinator enabled access to patient's medical records. Five sets of records, of most recently discharged or deceased patients in whose care the HPCT had participated, were made available, either on the ward or in the HPCT office. Coordinators made themselves available to answer auditor queries/questions at times throughout the visit.

Teams were asked to comment on the reports that they received following the audits.

Following the second pilot audit, the final draft consultation document was completed and disseminated to a total of 23 hospital palliative care team services in October 2002. The services consisted of the participating teams, those registered as THAG Users (interested in participating in the development of the standard/audit tools) and other units selected from the Hospice Information directory (2002).

The process of consultation, testing and review is designed to establish two aspects of validity: face validity (i.e. is the tool clear, logical and relevant?) and content validity (i.e. does the measure omit anything important?).

## RESULTS

The auditor identified 20 changes to the standard criteria, audit tools and auditing documentation from the first round of testing. Some common themes for change were identified from the majority of reports. Table 2

**Table 2:** First round piloting – recommendations for changes or additions to the standard criteria/audit tools

**Structure (S) criteria (numbered)**	**Process (P) criteria (numbered)**	**Audit tools**
S2: consider change to ‘The team has a written policy relating to its clinical role and function, including its place in cancer site-specific MDTs’	Current P3 and P4 should be interchanged. The current P9 should follow-on after P4, becoming P5	Add statement relating to ‘Information/clinical guidelines to Trust wards and departments – ‘evidence of the process of dissemination and evidence of date of future review’
		
S4: review wording. Consider, ‘team member’ rather than ‘nominated member’	P8 repeats much of criteria 7. Change to, ‘The HPCT has evidence of assessment of palliative care training needs for staff within the Trust’	Supplementary question to S5, ‘Evidence of Trust policies related to care of the dying or patients with chronic progressive illnesses’
Audit evidence should include job specification/ description for Service Manager/Head of Service	Once revised, this criterion should be interchanged with P7	
This criterion statement requires review regarding its relevance, or wording if appropriate		
		
S5: should be removed, as the criterion is more appropriately an outcome, and covered as such in O4		P1 to 3, develop a checklist of interventions undertaken (relevant to the role) to cover evidence required for these criteria
S7: add to wording, ‘and the collation of CMDS data’		P5: ‘Review of medical notes/HPCT documentation – for statements or records of discussions with ward team members’
		P6: ‘Review of role specification for HPCT clinical staff’ and ‘Review of HPCT Education Programmes, for supportive elements, such as handling difficult questions and breaking bad news’
		P5 & 6 should be framed as questions to staff; ‘Do you get feedback following the assessment by the HPCT member?’ and ‘Have there been times when you have found caring for a dying patient/family difficult, and have sought support from the HPCT? If yes, was the support helpful?’
		
	P12: is a structural criterion and would be covered by the revision to S7	P11: change wording to, ‘Evidence of audit data, reports and records of effect on practice’

There were no records for change regarding Outcome criteria.

illustrates the value of testing the tools, highlighting the gaps in criteria or the absence of specific audit questions. It also offers an insight into the process of standard development, showing how specific criteria change over a span of time, testing and review. There was substantial redrafting and reformatting of the standard package, with a further consultation round following on.

A further 10 changes to the standard criteria, audit tools and documentation were identified (Table 3

**Table 3:** Second round piloting – recommendations for changes or additions to the standard criteria/audit tools

**Structure (S) criteria (numbered)**	**Process (P) criteria (numbered)**	**Outcome (O) criteria (numbered)**	**Audit tools**
S2: this criterion is double-barrelled and should be split to allow identification of specific evidence	P10: add bullet points to include HPCT representation at cancer site-specific MDT meetings	O2: consider a change of wording to, ‘The HPCT uses a validated outcome measure to assess the effectiveness of specialist activity and interventions to meet patient's needs’	Documentation audit record – revise to reflect the order and evidence detail of both structural and process criteria
S4: change wording of criterion to ‘The team has systems, including documentation, for referral to and liaison with other specialist palliative care providers in the community and hospice/palliative care unit’. This clarifies the evidence required			Review of HPCT documentation and patient medical records audit record/Intervention record – rationalise and develop a more practical, landscape format record on a single A4 sheet
It will be helpful to teams to have examples of documentary evidence included in this criterion			
			
S5: as for S2, split to cover evidence for each area of documentation			Ward Staff Questionnaire: Q4 – add the wording ‘ during your time on the ward/In the past 2 years, etc’ and add a note to the auditor to ensure that the correct phrase is used
S9/10: create tick boxes for evidence relating to service details (S9) and aspects of management (S10)			

contains the details).

Table 4

**Table 4:** Summary of comments from the nine pilot sites on the audit reports for their services

**Question**	**Yes**	**No**	**Other**	**Comments**
Was the format of the report logical and effective?	× 8	× 1		Difficult to read. Constant referring back to standards.
Was the detailed evidence useful?	× 9			Extremely. Useful to have an objective review of our evidence
Were the recommendations relating to the evidence presented useful to the service?	× 9			Food for thought and very useful suggestions
				Raised awareness of issues
				But there need to be some additions
				
Did the observations relating to the audit tools/questions make sense and appear logical?	× 9			No comments received
Are there specific changes that you would like to see to the report content, format and layout?	× 3	× 6		Highlight areas done well
				Clearer action points
				Could perhaps be a little more user friendly, that is, layout with appropriate titles and use of bold
				
Do you wish to adopt the THAG package as your regular audit tool?	× 7		Probably × 1	No comments received
			Possibly × 1	
				
Would your team feel able to act in a peer review relationship with another service?	× 8		Probably × 1	No comments received
It is planned to review and revise (where appropriate) the HPCT standard annually. Would you wish to participate in this review?	× 7		No comment × 2 services	No comments received
			Not on an annual basis × 1 service	

illustrates the range of comments from the participating pilot sites on the format and content of their individual audit reports.

An indication of the relevance and acceptability of the audit tool is confirmed by the observation that when the pilot HPCTs were asked whether they would like to accept THAG as their quality measure of choice, the majority (seven of nine) stated that they would, while the remaining two services indicated ‘probably’ and ‘possibly’ to the question.

The sample, though a small proportion of HPCT services, covered a wide geographical spread, from the North-West to the South-East of England. Responses to the preparation for testing, and the feedback following audit reporting, strongly suggest that the measure criteria were relevant, reasonable and acceptable to HPCTs (face validity).

Although the aim of the pilot audits was to validate and refine the audit tools, the results of the audit were made available to teams for their use. Some examples are included here to illustrate the types of information that can be obtained:

### Workload/casemix

Of 87 sets of HPCT documentation reviewed, the levels of intervention were recorded in 49% (43/87); 82 sets of medical records were reviewed overall. The range of HPCT recorded visits to patients, across the pilot sites, was 1–32 per patient.

Reasons for referral and continued support were consistent across the pilot sites and covered the range of HPCT activity. However, there were some developments in activity, which reflected pressure on ward staff, rather than a natural extension of the role of the HPCT, for example, liaison with other professionals and discharge planning. Here, HPCT staff appeared to have taken on total responsibility for these aspects of care, with ward-based representatives indicating that this was what they expected from the specialist resource.

### Ward staff views

‘[They are a] good coordinator of care, organising family meetings and appropriate discharge plans’, and ‘[They help] patients and families come to terms with what's happening, we don't have the time to do that stuff, can't organise complex discharges’.

Ward staff comments on the supportive role of the HPCT for patients, families and professionals were consistent across the pilot sites.

### Effectiveness of education programmes

Of the 36 ward staff interviewed overall, 78% had not attended an HPCT educational programme in the previous 2 years.

Three pilot sites presented completed modified THAG Education Evaluation Questionnaires as evidence. The questionnaire asks ward staff (predominantly nurses) to identify the education programme, offered by the HPCT, that they have attended within the previous 2 years.

Staff were asked to comment on the improvements in their knowledge and skills in palliative care as a result of attending the study event. They were also asked to state whether any area of their practice had changed following the educational programme. The response rates in the three areas were 13% (four out of 30); 69% (34 out of 49) and 25% (six out of 24) respectively. Combining the results from the three areas 61% (27 out of 44) respondents identified an improvement in knowledge, 59% (26 out of 44) an improvement in skills, and 43% (19 out of 44) a change in palliative care practice.

Two pilot sites were using a PACA record in their documentation. However, there was either a single entry record or the frequency of recording did not reflect the number of visits to the patient.

## DISCUSSION

The principal aim of testing the audit tools was achieved. It enabled us to refine them, particularly the documentation for completion by the auditor. Comments from services on the criterion statements were incorporated into subsequent revisions.

This has established face and content validity of the standards and audit tools.

The example results from the pilot audits helped to describe the workload and casemix of an HPCT, ward staff views of the team and the effectiveness of education programmes provided by the team. For individual teams these results not only provide a measure of quality assurance but also facilitate a review of the service and inform future service developments.

The recent consultation has also raised a number of areas that require further consideration, including telephone advice activity and its recording as workload, and the need for an HPCT to actively engage with its palliative care network. A more significant issue is that of assessing whether an HPCT is adequately resourced to support its hospital population, acknowledging the differing dependency of patients.

Clearly, it is important to continue the development of a range of measures to monitor the effectiveness of hospital-based palliative care. A recent systematic literature review, to determine whether hospital-based palliative care teams improve the process or outcomes of care for patients and families at the end of life (Higginson *et al*, 2002), recommended that future evaluations should:

- ‘Compare different models of hospital based team, namely those giving a more intensive intervention *vs* those that are more advisory and concentrate on education and have lower contact with patients.
- Use standardised outcome measures assessing patient pain, symptoms, carer outcomes and, where possible, the effect on professionals or the overall hospital service’ (p 104).

The revised version of the national Manual of Cancer Standards is due to be published in early 2004. The new specialist palliative care standards are likely to reflect the recommendations of the NICE Guidance on Supportive and Palliative Care for Cancer, which is also due to be published in early 2004. They will probably be of similar style to those from the first version of the Manual, that is, will largely focus on structure with some process standards.

The standards and audit tools reported here cover a wide range of aspects of HPCT work and we feel provide a useful framework for HPCTs to quality assure, review and develop their services. It is likely that they will complement the new National Standards when they become available.

Information on obtaining the THAG ‘Hospital Palliative Care Team Package’ is available from: Helen Crisp, Health Quality Service, 15 Whitehall, London, SW1A 2DD, Tel: 020 7389 1001, E-mail: hcrisp@hqs.org.uk
